# Fusion Between Control Mesoangioblasts and mtDNA-Mutant Myotubes Preserves Myotube Morphology and Mitochondrial Network Organization

**DOI:** 10.3390/ijms27031357

**Published:** 2026-01-29

**Authors:** Somaieh Ahmadian, Patrick J. Lindsey, Monique Ummelen, Anton Hopman, Marc A. M. J. van Zandvoort, Hubert J. M. Smeets, Florence H. J. van Tienen

**Affiliations:** 1 Department of Translational Genomics, Maastricht University Medical Centre+, 6229 ER Maastricht, The Netherlands; 2 GROW Research Institute for Oncology and Reproduction, Maastricht University, 6229 ER Maastricht, The Netherlands; 3 Department of Genetics and Molecular Cell Biology, Maastricht University, 6229 ER Maastricht, The Netherlands; 4 Institute for Mental Health and Neurosciences (MHeNS), Maastricht University Medical Centre+, 6229 ER Maastricht, The Netherlands; 5 Cardiovascular Research Institute Maastricht (CARIM), Maastricht University, 6229 ER Maastricht, The Netherlands; 6 Institute for Molecular Cardiovascular Research (IMCAR), Universitätsklinikum Aachen, 52074 Aachen, Germany

**Keywords:** mtDNA mutation, mesoangioblasts, mitochondrial membrane potential, mitochondrial mass

## Abstract

Mitochondria are the energy factories of a cell and mitochondrial morphology, quantity, membrane potential, and DNA copy number can change depending on metabolic requirements and/or genetic defects. Different mutations in mitochondrial DNA might affect mitochondrial morphology and membrane potential differently. In this study we investigated mitochondrial morphology and membrane potential in vitro in mesoangioblast-derived human myotubes harboring a pathogenic mtDNA mutation and analyzed mitochondrial behavior following fusion with healthy mesoangioblasts. Myotubes were differentiated in vitro from mesoangioblasts obtained from two mitochondrial myopathy patients, M02 (96% m.3271T>C) and M11 (73% m.3291T>C), and from a functionally healthy male control, M06 (3% m.3243A>G). On day 5 of differentiation, healthy male mesoangioblasts (mM06) were added to mutant myotube cultures to allow cell fusion. On day 11, mitochondrial morphology and membrane potential were assessed by three-dimensional live-cell imaging using spinning disk confocal microscopy with tetramethylrhodamine methyl ester (TMRM). Following live imaging, cells were fixed and subjected to Y-chromosome fluorescence in situ hybridization (FISH), enabling identification and retrospective analysis of hybrid (i.e., fused with male control mesoangioblasts) and non-hybrid (i.e., not fused with these control mesoangioblasts) myotubes within the same imaging fields. Quantitative image analysis at the level of individual myotubes revealed that, when normalized to sarcoplasmic volume, mitochondrial volume, object number, and membrane potential did not differ between mutant and control myotubes despite heteroplasmy levels exceeding 70%. Fusion of healthy mM06 mesoangioblasts did not impair myotube formation and resulted in redistribution of mitochondrial content without an increase in mitochondrial object number, consistent with integration of donor mitochondria into the existing mitochondrial network. Across conditions, mitochondrial parameters were strongly influenced by myotube size, underscoring the importance of accounting for biological variation when quantifying mitochondrial features. Together, these findings demonstrate that high mtDNA mutation loads do not necessarily alter mitochondrial morphology or membrane potential under standard in vitro differentiation conditions and provide mechanistic insight into mitochondrial behavior following mesoangioblast fusion in human myotubes. Fusion of healthy mesoangioblasts supports integration of donor mitochondria into the existing network without compromising myogenesis, consistent with mitochondrial mixing rather than replacement.

## 1. Introduction

Mitochondria are essential organelles for energy production in eukaryotic cells. Their function is regulated by both nuclear DNA and mitochondrial DNA (mtDNA), which encodes key components of the respiratory chain and ATP synthase, as well as RNA molecules required for mitochondrial protein synthesis. Each cell contains multiple copies of mtDNA. The default is homoplasmy, which means that the mtDNA is the same in all, but mutated and wildtype mtDNA copies can also coexist within the same cell, which is known as heteroplasmy. In cases of mitochondrial disease due to a heteroplasmic mtDNA mutation, symptoms typically arise when heteroplasmic mutation load exceeds a tissue-specific threshold. High energy-demanding tissues like brain and skeletal muscles are most commonly affected [1]. Patients with mitochondrial myopathy suffer from fatigue, muscle weakness, and exercise intolerance [2]. Jeppesen et al. showed in a study with 50 m.3243A>G mutation carriers that all carriers with <50% m.3243A>G mutation in skeletal muscle showed no exercise intolerance, while part of the 50–80% mutation load carriers and most mutation carriers with >80% mutation load did show exercise intolerance, showing a clear relationship between m.3243A>G mutation load and skeletal muscle functioning [3]. Currently, there is no effective cure for mitochondrial myopathies, and patients are supported with symptomatic management by receiving dietary supplements, medication for muscle pain, physical therapy, and respiratory support [4].

To combat mitochondrial myopathy caused by an mtDNA mutation, our group is developing a therapy using autologous muscle stem cells, called mesoangioblasts, with a low mtDNA mutation load. Mesoangioblasts can be efficiently isolated from skeletal muscles and expanded ex vivo till sufficient numbers for systemic administration to treat all muscles. Upon intra-arterial delivery, mesoangioblasts migrate to damaged skeletal muscles where they can form new fibers or fuse with existing muscle fibers. Preclinical studies demonstrated that mesoangioblasts of about half of analyzed mtDNA mutation carriers displayed a low mtDNA mutation load and fulfilled all criteria for therapeutic application, allowing transplantation of autologous mesoangioblasts [5]. The first in-human clinical study demonstrated that one intra-arterial administration of autologous mesoangioblasts in the lower leg of m.3243A>G mutation carriers is safe [6], and a currently ongoing phase IIa clinical study aims to assess the effect of three mesoangioblast administrations on muscle strength, exercise intolerance, and m.3243A>G mutation load in the arm of m.3243A>G patients with a measurable functional deficit.

In a previous in vitro study, we have reported that the addition of healthy mesangioblasts to myotubes with a high mtDNA mutation load leads to a proportional reduction in mtDNA mutation load [7]. Therefore, the current in vitro study assesses possible changes in mitochondrial morphology and membrane potential in myotubes of mtDNA patients and the effect of fusion with healthy mesoangioblasts. To this end, we used confocal spinning disk microscopy in combination with mitochondrial staining using tetramethylrhodamine methyl ester (TMRM), as described before [8], to study myotubes derived from mesoangioblasts of two female mtDNA mutation carriers (M02 with 96% m.3271T>C and M11 with 73% m.3291T>C) and one male control without the m.3271T>C or m.3291T>C mutation.

## 2. Results

First, we determined mitochondrial volume, number of individual mitochondrial objects, and membrane potential in non-hybrid myotubes, i.e., directly derived from 11 days differentiated mesoangioblasts with a high mtDNA mutation load (M02 and M11) and a control (M06) to assess a possible effect of the high mtDNA mutation load. Next, we assessed the effect on these parameters following addition of control male mesoangioblasts (mM06) at differentiation day 5 to female myotubes of M02 and M11 with an mtDNA mutation, i.e., hybrid myotubes. These analyses were performed in one single experiment which contained both hybrid and non-hybrid myotubes.

### 2.1. Analysis of Myotube Fusion Index

First, the Myotube Fusion Index (MFI) was assessed (Table 1). MFI was calculated from eight fields of view per culture for non-hybrid (M02, M11, and M06) and hybrid myotubes (M02+mM06 and M11+mM06). The fusion index was defined as the percentage of nuclei located within multinucleated myotubes relative to the total number of nuclei per field of view.

In addition, we compared if there were significant differences in the number of nuclei or in sarcoplasm volume per myotube between these cell cultures. One-way ANOVA of these variables showed no statistically significant differences (*p* > 0.05) within the non-hybrid myotubes. Next, hybrid and non-hybrid cell culture pairs were analyzed using *t*-test. While no significant changes on MFI were observed, addition of mM06 to M02 myotubes resulted in myotubes with significantly increased (*p* < 0.05) number of nuclei/myotube, sarcoplasm volume/nucleus, and total sarcoplasm volume per myotube (Table 1). In contrast, mM06 fusion with M11 only significantly increased the number of nuclei/myotube and sarcoplasm volume/nucleus, but not total myotube sarcoplasm volume.

As analyzed myotubes contained varying numbers of nuclei per myotube (range 3–36), which was associated with varying sarcoplasm volume, this needs to be corrected to enable analysis of mitochondrial parameters. To compare non-hybrid M02, M11, and M06, we first verified that the sarcoplasm volume increases proportionally with the number of nuclei per myotube. This was supported by a strong linear relationship (R^2^ values between 0.78 and 0.89), intercepts that were not significantly different from zero, and less than 10% variation in slope between the three cell cultures (Supplemental Figure S2). This indicates that in non-hybrid cells, mitochondrial parameters can be normalized to either myotube sarcoplasm volume or number of nuclei per myotube. However, in hybrid myotubes, 33% variation between slopes of M02 and M02+mM06 (y = 4025.5x vs. y = 5342.9x) and between slopes of M11 and M11+mM06 (y = 4157.9x vs. y = 2773.2x) was observed. This substantial variation excludes normalization of mitochondrial parameters to sarcoplasm volume in these hybrid myotubes. Therefore, the number of nuclei was used for analysis of hybrid myotubes and the sarcoplasmic volume and/or number of nuclei was used for analysis of the non-hybrid myotubes.

### 2.2. Analysis of Mitochondrial Morphology and Membrane Potential in Non-Hybrid Myotubes with High Mutation Load (M02, M11) and a Healthy Control (M06)

**Figure 1:**
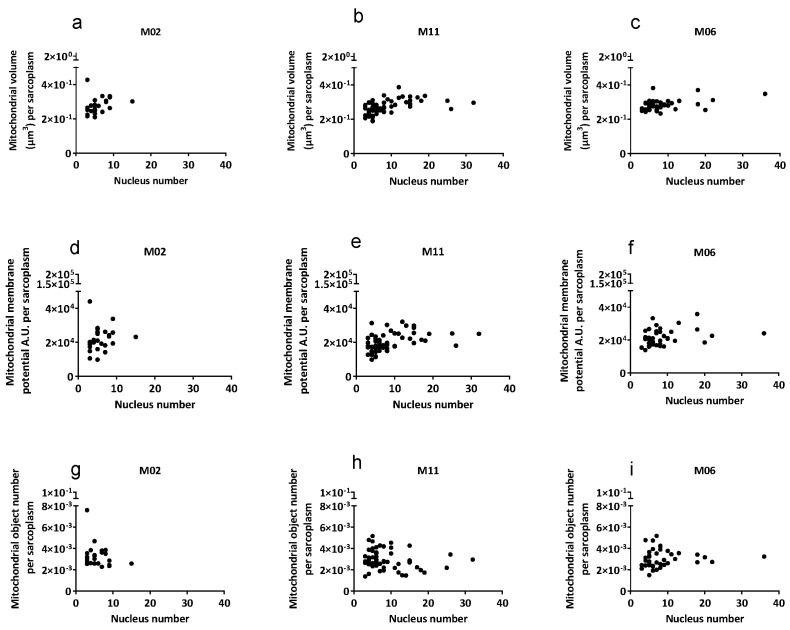
Mitochondrial characteristics in non-hybrid myotubes derived from mesoangioblasts with high mutation load (M02, M11) and control (M06). (**a**–**c**) Mitochondrial volume (µm^3^) per myotube normalized to sarcoplasmic volume (µm^3^) is plotted; (**d**–**f**) Mitochondrial membrane potential (A.U.) of a myotube normalized to sarcoplasmic volume (µm^3^), and (**g**–**i**) mitochondrial object number per myotube normalized to sarcoplasm volume (µm^3^). Each data point represents a single analyzed myotube. The x-axis indicates the number of nuclei per myotube. Individual myotubes analyzed per cell culture were *n* = 25 (M02), *n* = 57 (M11), and *n* = 41 (M06).

First, total mitochondrial volume and organization (number of individual mitochondrial objects) was analyzed in myotubes derived from mesoangioblasts with a high mutation load (M02, M11) and from control mesoangioblasts (M06). The parameters were mitochondrial volume (µm^3^) per myotube, mitochondrial membrane potential (A.U.), and the number of mitochondrial objects per myotube normalized to myotube cytoplasm volume (sarcoplasm). As shown in Table 2 and Figure 1, when corrected for sarcoplasm volume, no significant differences were apparent in mitochondrial volume, mitochondrial membrane potential, and mitochondrial object number between myotubes of three different cell cultures. Given the differences in size among individual myotubes, mitochondrial parameters were normalized to sarcoplasmic volume and plotted against the number of nuclei per myotube (Figure 1). After normalization, mitochondrial parameters remained comparable across myotubes with increasing nucleus number, indicating proportional scaling with myotube size rather than changes in mitochondrial function per unit sarcoplasm.

### 2.3. Hybrid Myotubes: Fusion of Control Mesoangioblasts with Myotubes with High mtDNA Mutation Load

**Figure 2:**
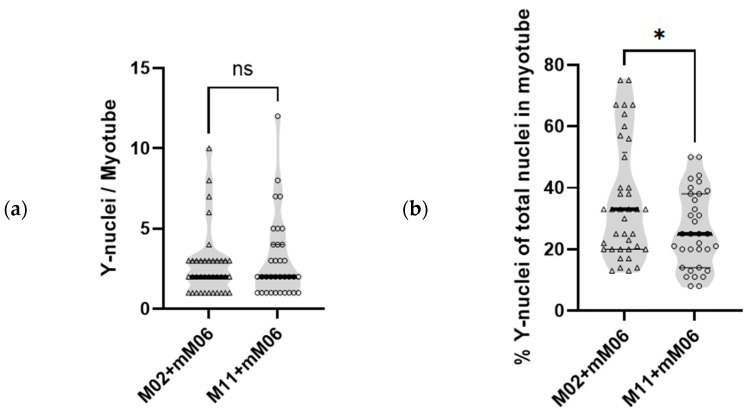
Contribution of control mesoangioblasts with Y-chromosome-positive nuclei to mutant myotubes. (**a**) Violin plot showing the number of control mM06 mesoangioblasts (Y-nuclei) in the M02+mM06 and M11+mM06 hybrid myotubes. (**b**) Violin plot showing the proportion (percentage) of Y-positive control mesoangioblast nuclei divided by the total number of nuclei within each myotube. Individual data points represent single myotubes; triangles indicate M02+mM06 myotubes and circles indicate M11+mM06 myotubes. Individual myotubes analyzed per cell culture were *n* = 38 for M02+mM06 and *n* = 34 for M11+mM06. Statistical analysis was performed using an unpaired *t*-test. * indicates *p* < 0.05; ns: not significant.

As shown in Table 1, hybrid myotubes (M02+mM06 and M11+mM06) contained on average 2.2 and 3.6 more nuclei per myotube, respectively, compared with non-hybrid M02 and M11 myotubes, corresponding to increases of 38% and 39%. As mM06 mesoangioblasts are derived from a male donor, their nuclei contain a Y-chromosome, which was fluorescently labeled to enable quantification of the contribution of mM06 mesoangioblasts to each individual hybrid myotube. As shown in Figure 2a, the total number of Y-chromosome nuclei from mM06 per myotube was not significantly different in both groups of hybrids. However, due to non-hybrid M02 having less nuclei/myotube than non-hybrid M11 (5.8 ± 2.8 vs. 8.8 ± 6.1, mean ± SD, respectively), the relative contribution of control mM06 mesoangioblasts (Y) fused per total number of nuclei in the hybrid M02+mM06 myotubes is larger. As shown in Figure 2b, the percentage of Y-nuclei in M02+mM06 hybrid myotubes was 35.5% ± 18.9% (mean ± SD, range: 12.5–75%), while M11+mM06 myotubes showed a significantly lower percentage of Y-nuclei 26.2% ± 12.3% (mean ± SD, range: 8.3–50%).

### 2.4. Analysis of Mitochondrial Morphology and Membrane Potential in Hybrid Myotubes Formed by Fusion of High mtDNA Mutation Load Myotubes with Control Mesoangioblasts

Hybrid M02+mM06 and M11+mM06 myotubes were compared with their respective non-hybrid counterparts (M02 and M11) to evaluate the contribution of control mM06 mesoangioblasts to mitochondrial properties following fusion with mtDNA-mutated myotubes. Mitochondrial parameters were calculated per myotube volume, per μm^3^ sarcoplasm volume, and per myotube nucleus (Table 3). However, as previously explained, the slope of the relationship between sarcoplasm volume and nuclei number differed significantly between hybrid and non-hybrid cultures (Supplementary Table S1); therefore, we considered normalization per nucleus to be most appropriate.

**Figure 3:**
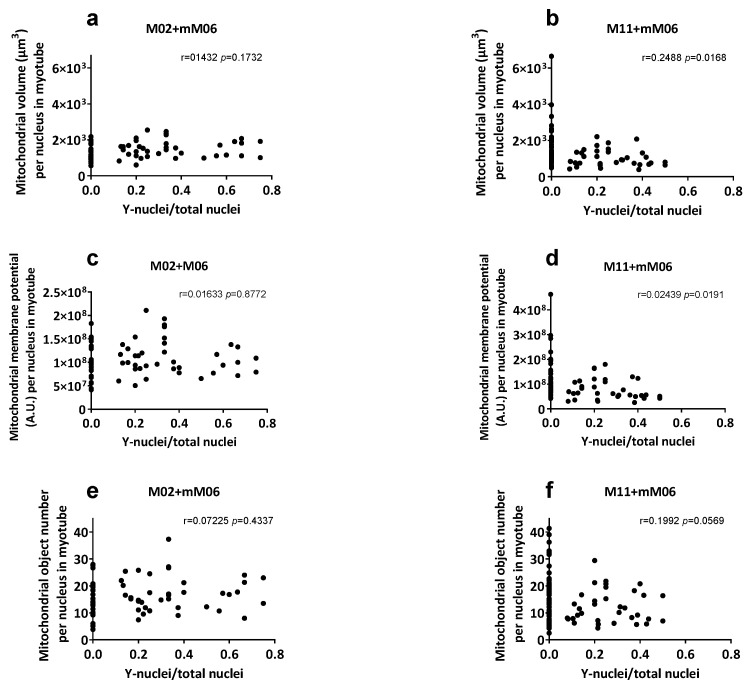
Mitochondrial characteristics of mutant myotubes (M02, M11) and hybrid myotubes generated by fusion of mutant myotubes with control mesoangioblasts (mM06). Mitochondrial volume (µm^3^) per myotube nucleus (**a**,**b**), mitochondrial membrane potential (TMRM intensity, A.U.) per myotube nucleus (**c**,**d**), and the number of mitochondrial objects per myotube nucleus (**e**,**f**) are shown on the y-axis. Data are plotted against the ratio of fused control nuclei (Y-positive) to the total number of nuclei per myotube (N) on the x-axis for fused myotubes (M02+mM06 (**a**,**c**,**e**); M11+mM06 (**b**,**d**,**f**)). The number of individual myotubes analyzed per condition: M02 *n* = 25; M02+mM06 *n* = 38; and M11 *n* = 57; M11+mM06 *n* = 34.

In comparisons between M11 vs. M11+mM06 hybrids, no differences were detected in total myotube volume, membrane potential, or number of mitochondrial objects when normalized per myotube or per sarcoplasm volume. When correcting for nuclei number, both mitochondrial volume and membrane potential were significantly reduced in M11+mM06, indicating reduced contribution of M06 mitochondria (Table 3). Analysis of these parameters relative to the proportion of mM06 nuclei within hybrid myotubes (Figure 3) showed a significant negative correlation in M11+mM06 for mitochondrial volume and membrane potential. In M11+mM06 hybrid myotubes, the number of mitochondrial objects per myotube nucleus was reduced, whereas the total number of mitochondrial objects per myotube remained unchanged, suggesting fusion of mM06-derived mitochondria with M11 mitochondria.

In contrast, M02+mM06 hybrids displayed a significant increase in mitochondrial volume (FC 1.70), membrane potential (FC 1.57), and number of mitochondrial objects (FC 1.55) per myotube, together with a significantly larger sarcoplasm volume (FC 1.72). When normalized per myotube nucleus, only total mitochondrial volume remained significantly increased by 1.15-fold, and no change was seen per sarcoplasm volume, indicating regulation by sarcoplasm volume.

**Figure 4:**
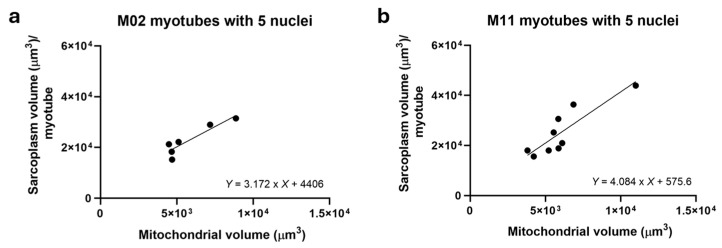
Relationship between mitochondrial volume and sarcoplasmic volume in non-hybrid myotubes containing five nuclei. (**a**) M02 (*n* = 7); (**b**) M11 (*n* = 9).

To further explore the relationship between mitochondrial volume and sarcoplasmic volume, non-hybrid M02 (*n* = 7) and M11 (*n* = 9) myotubes containing five nuclei were analyzed. As shown in Figure 4, mitochondrial volume strongly correlated with sarcoplasm volume in both, supporting the conclusion that sarcoplasm volume governs mitochondrial volume.

## 3. Discussion

In this study, we investigated how pathogenic mtDNA mutations affect mitochondrial morphology and membrane potential in mesoangioblast-derived myotubes, and examined the behavior of mitochondria following fusion with healthy mesoangioblasts. By using a combination of live-cell imaging and FISH techniques, we were able to track and analyze specific myotubes that had fused with healthy mesoangioblasts. We first studied mitochondrial morphology and membrane potential of mesoangioblast-derived myotubes of M02 with 96% m.3271T>C and M11 with 73% m.3291T>C and M06 with 3% m.3243A>G as a healthy control. The results from this study showed that mitochondrial morphology (total mitochondrial volume and number of individual mitochondrial objects) and membrane potential were similar across myotubes derived from both high mtDNA mutation load (M02, M11) and healthy control (M06) mesoangioblasts when normalized to sarcoplasm size. Normalization to sarcoplasm size was necessary for valid comparisons across cell cultures, as sarcoplasm volume correlated with the number of nuclei and as we compared myotubes with varying nuclear content. After correction, no statistically significant differences in mitochondrial volume, membrane potential, or mitochondrial object number were observed between the groups. Although M02 myotubes had a lower nucleus count compared to M11 and M06, this did not translate into differences in mitochondrial parameters after normalization. These findings indicate that, despite the presence of pathogenic mtDNA mutations at levels exceeding 70%, the baseline mitochondrial characteristics of in vitro differentiated myotubes do not differ from those of healthy controls.

Baseline differences were expected because morphological and biochemical abnormalities were reported for both mutations. The m.3271T>C mutation in MT-TL1, encoding mitochondrial tRNA^Leu(UUR), typically impairs mitochondrial translation and complex I activity, often reflecting reduced membrane potential and abnormal cristae in sensitive cell types when heteroplasmy exceeds ~70% [9]. Similarly, m.3291T>C, also affecting MT-TL1, impairs translation of subunits of the mitochondrial respiratory chain complexes, particularly complex I, III, and IV [10]. This causes complex I and III deficiencies, morphological abnormalities, and a decrease in membrane potential. In our study, despite >80% mutation load, we did not observe any change in mitochondrial morphology and membrane potential. Several factors may explain the absence of detectable differences. First, analysis at the level of individual hybrid myotubes necessitated the use of imaging-based approaches. Mitochondrial membrane potential was therefore assessed using TMRM as a functional readout linked to ATP production. Other mitochondrial functional assays would be informative but are not technically feasible in individual myotubes or cannot be performed on the same cell. Second, the choice for the spinning disk confocal microscopy was dictated by the need to image in living cells without any photodamage. However, as a drawback, this imaging approach is not sensitive enough to identify subtle subcellular structural changes in mitochondrial cristae architecture. This is supported by Stephan et al. [11] and Ren et al. [12], who demonstrated that super-resolution microscopy (e.g., STED nanoscopy) is required to visualize fine cristae changes in mitochondria. Therefore, we could only look at more general characteristics such as volume, number of mitochondrial objects, and mitochondrial membrane potential. Third, the timing of measurements (day 11 of differentiation) may be too early to observe pronounced mitochondrial dysfunction, which often becomes evident under prolonged differentiation or metabolic stress [13]. Myotubes at this stage may not have reached full contractile or metabolic maturity. Immature myotubes often lack organized sarcomeres and exhibit limited contraction activity, which reduces their energetic demand [14]. As mitochondrial membrane potential is closely tied to ATP turnover, this reduced metabolic activity may contribute to the absence of detectable depolarization despite high mutation load. Fourth, the culture conditions, specifically usage of high glucose medium, might introduce insufficient metabolic stressors, such as nutrient deprivation or high energy demand, which are known to unmask mitochondrial defects in high-heteroplasmy models [15,16]. As no mitochondrial abnormalities were observed, we could not test if the fusion would lead to the expected improvement of mitochondrial function. However, still a number of interesting observations were made.

First, we showed that fusion of control mM06 mesoangioblasts with mutant myotubes did not have a negative effect on myotube formation, as MFI did not significantly differ between non-hybrid and hybrid conditions of M02 or M11 cell cultures. Secondly, changes in mitochondrial volume and membrane potential of hybrid myotubes were observed. M02+mM06 hybrid myotubes displayed a significant increase with respect to all analyzed mitochondrial parameters and increased sarcoplasm volume. When calculating mitochondrial volume per nucleus, a significant increase of 1.15-fold is observed, while no significant change is observed when calculating mitochondrial volume per sarcoplasm volume, indicating that mitochondrial volume is controlled by the sarcoplasm volume. In line with this, in [17], resistance training was found to result in a reduction in both mitochondrial volume as well as sarcoplasmic volume in biceps brachii muscle of previously untrained individuals. The increases in mitochondrial parameters observed in M02+mM06 hybrids were not observed in M11+mM06 hybrid myotubes. This can likely be explained by the cell size, as non-hybrid M06 myotubes are substantially bigger (~17%) with respect to sarcoplasm volume and mitochondrial volume per nucleus compared to M02, while being similar to M11. Fusion of mM06 to M11 rather resulted in a proportional reduction in mitochondrial volume and membrane potential when corrected for myotube nuclei number. This is likely the consequence of different maturation stages, as M11 was differentiated for 11 days, while mM06 was added on day 5 and only had the opportunity to differentiate for a maximum of 6 days. To verify this, longer myogenic differentiation is required, which is not feasible in 2d culture, but could be achieved using 3d tissue-engineered myogenic culture [18]. Taken together, our data demonstrated a strong relation between mitochondrial content and sarcoplasm volume, which should be taken into consideration when analyzing mitochondrial parameters.

Thirdly, after fusing mM06 mesoangioblasts with M11 myotubes, the number of mitochondrial objects per nucleus is reduced, while the total number of mitochondrial objects per myotube is not changed compared to M11, indicating integration and formation of bigger mitochondrial networks. In line with this, the number of mitochondrial objects per myotube nucleus is unchanged in M02+mM06 hybrid myotubes, despite a 1.15-fold increase in mitochondrial volume per nucleus. This data suggests fusion of the mM06-derived mitochondria with M11/M02 mitochondria, which is obviously something we would like to achieve in our therapy. Our prior work with Zelissen et al. demonstrated a proportional reduction in mtDNA mutation load after fusion [7] and the current data suggest that this is a fusion with existing mitochondria and not an addition of healthy ones. This is consistent with previous studies demonstrating that mitochondria fuse following cell–cell fusion or nanotube-mediated mitochondrial transfer, with the resulting mitochondrial population being stably propagated [19,20].

## 4. Methods and Material

### 4.1. Cell Culture

Mesoangioblast isolation and culture were performed as described before [5]. Mesoangioblasts were cultured between passage number 4 and 15 in a 37 °C humidified incubator in 4% O_2_ and 5% CO_2_, using IMDM medium supplemented with 10% fetal bovine serum (Bodinco, Alkmaar, The Netherlands), 1× glutamine, 1× sodium-pyruvate, 1× non-essential amino acids, 1× insulin transferase selenium X, 0.2% 2-mercaptoethanol, 5 ng/mL FGF2 (Miltenyi Biotec, Teterow, Germany), and 0.1% gentamycin. To allow later spinning disk confocal microscopy of myotubes, mesoangioblasts (25,000 cells/cm^2^) were subsequently seeded on a 1:27 dilution of Matrigel (hESC-qualified Matrix (5 mL LDEV-free))-coated 4-well microscopy µ-slides (Ibidi). All three mesoangioblast cultures were subsequently differentiated to myotubes by allowing them to reach 100% confluence and at day 0 replacing mesangioblast growth medium with myogenic differentiation medium, which consists of DMEM containing 2% horse serum. At day 5 after starting myogenic differentiation, mesoangioblasts (20,000 cell/cm^2^) from the healthy male control, (mM06), resuspended in DMEM 2% horse serum were added to initiate fusion of these cells with the M02/M11 forming myotubes. At day 11, cells were stained with 200 nM tetramethyl rhodamine methyl ester (mitochondria, red, TMRM), 2 μM Calcein AM (cell cytoplasm, green), and 2 μM Hoechst 34580 (Cell nuclei, blue) in medium for 30 min at 37 °C and 5% CO_2_. After incubation, medium was replaced with fresh differentiation medium and live imaging was performed using spinning disk confocal microscope as described below. All materials in this study were purchased from ThermoFisher Scientific (Waltham, MA, USA) unless stated otherwise.

### 4.2. Confocal Imaging of Mesoangioblasts and Myotubes and Image Analysis

Myotubes were imaged using CorrSight SDCM using a Zeiss 63× oil immersion (White Plains, NY, USA) with numerical aperture (NA) 1.3. Myotubes were imaged using a 20× air objective (Waltham, MA, USA) with NA 0.9. The microscope was equipped with an Andromeda spinning disk module, and a Hamamatsu ORCA-Flash 4.0 V2 camera (Hamamatsu, Japan) with a laser light source that emits at different wavelengths of 405, 488, 561, and 640 nm. Excitation at 561 nm was used for acquiring images for mitochondria stained with TMRM (red), excitation at 488 was used to image the cytoplasm stained with Calcein–AM (green), and excitation at 405 nm was used to image the nuclei stained with Hoechst 34580 (blue). Emission filters FF01 593/46-25, FF01 523/30-25, and FF01 446 nm were used, respectively. Live-cell imaging of mesoangioblasts and myotubes was carried out inside a 37 °C and 5% CO_2_ controlled chamber (Ibidi). For live-cell imaging of myotubes, 150 Z-stacks were acquired. All images were deconvoluted using Huygens Professional version 21.10 (SVI) using the Classic Maximum Likelihood Estimation algorithm with a signal-to-noise ratio of 7.0, maximum iterations of 40, and quality threshold of 0.001.

### 4.3. FISH and Imaging of Fixed Samples

To identify male control mesoangioblast (mM06) nuclei in myotubes of female myotubes (M02/m11), FISH staining of Y-chromosome was used. Immediately after live-cell imaging of the myotubes, cells were fixed in 4% formaldehyde in PBS for 5 min, then permeabilized in 0.2% Triton X-100 in PBS for 30 min, dehydrated with 70, 96, 100% Ethanol. Subsequently, 5 ng/µL of the Y-chromosome probe (DYZ3 probe: targets Y-chromosome centromeric DNA [21]) conjugated to Y-ATTO 550 dissolved in 10% dextran sulfate and 60% formamide and 2XSSC (1 xSSC is 0.15 M NaCl, 0.015 M sodium citrate, pH 7.0) was added to each well of the plate. The plate was placed for 10 min on a preheated hotplate at 80 °C, after which in situ hybridization was allowed overnight in 37 °C incubator, followed by PBS washes and 1 µg/mL DAPI staining on the following day. Using the DAPI and Y-ATTO 550 channels, the same field of views of the fixed samples were imaged using SDCM and the previously recorded live images of myotubes were overlayed. The distinction between hybrid (M02+mM06, M11+mM06) and non-hybrid (M02, M11) myotubes could now be made based on the presence of the stained Y-chromosomes in the overlay images.

### 4.4. Analysis

**Figure 5:**
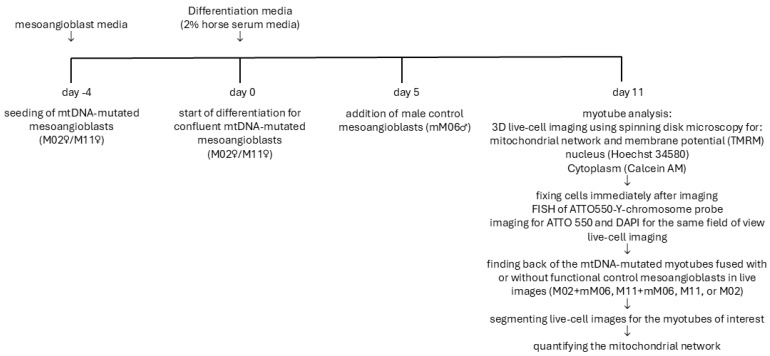
Schematic representation of the study design for mitochondrial network analysis and Y-chromosome localization in mesoangioblasts.

Analysis of the mitochondrial network was performed manually on the segmented images of the fused myotubes using a script in Matlab R2021a (The Mathworks, Inc.) with additional DipImage toolbox (TU Delft) developed previously in our group, which was adopted from Koopman group [22]. Deconvoluted images were filtered both with Median 3D filter and top-hat and masked using thresholding at a constant value as described in [8,22]. Cytoplasm volume was calculated by subtracting the hole-filled nuclear volume using the Hoechst staining from the hole-filled cell volume defined by Calcein AM staining. The full schematic representation of the study design is presented in Figure 5 and presents a schematic representation of the experimental workflow. It outlines the differentiation of mesoangioblasts into myotubes, the fusion of male control mesoangioblasts with both control and mtDNA-mutant myotubes, live imaging for mitochondrial morphology and membrane potential, and FISH analysis to distinguish hybrid from non-hybrid myotubes. Supplementary Figure S1 illustrates the process of identifying fused control mesoangioblasts within mutated myotubes. Live-cell imaging visualized mitochondrial networks (TMRM), nuclei (Hoechst), and cytoplasm (Calcein AM). FISH using a Y-chromosome-specific probe confirmed fusion by detecting male nuclei within female-origin myotubes. Segmentation of live images allowed classification of mitochondrial structures and membrane potential. Mitochondria in fused myotubes were segmented and color-coded to represent discrete objects, and TMRM intensity maps provided qualitative assessment of membrane potential.

### 4.5. Myotube Fusion Index

The Myotube Fusion Index (MFI) represents the proportion of nuclei incorporated into multinucleated myotubes compared to the total nuclear count within an observed microscopic field. This metric is expressed as a percentage, calculated by dividing the count of nuclei found within myotubes by the total number of nuclei present in the field of view and multiplied by 100%.

### 4.6. Statistical Analysis

All data were initially checked for normality using the D’Agostino and Pearson test. Once normality was confirmed, comparisons among three groups were conducted using ordinary one-way ANOVA. To identify specific differences between groups, Tukey’s multiple comparisons test was applied as a post hoc analysis. For comparisons between two groups, an unpaired *t*-test was used. Analysis was performed by GraphPad Prism 8.4.

## 5. Conclusions

Our study shows that, despite the presence of a pathogenic mtDNA mutation at heteroplasmy levels exceeding 70%, the mitochondrial volume, object number, and membrane potential of in vitro differentiated myotubes under standard culture conditions do not differ from those of healthy controls. Fusion of healthy mesoangioblasts with mutant myotubes demonstrated integration of donor mitochondria into the existing mitochondrial network. In line with our previous observation of proportional mtDNA mutation load reduction following mesoangioblast fusion, these findings support a therapeutic mechanism based on mitochondrial fusion and mixing with resident mitochondria, thereby reinforcing the translational potential of mesoangioblast-based strategies for mtDNA-associated myopathies. Furthermore, our data demonstrates that fusion does not influence myogenic capacity and emphasizes the importance of taking into account biological variation in cell size when quantifying mitochondrial parameters.

## Figures and Tables

**Table 1:** Myotube Fusion Index (MFI) and number of nuclei in analyzed hybrid and non-hybrid myotubes.

	M02 Mutant Myotubes (*n* = 25)	M02+mM06 Hybrid Myotubes (*n* = 38)	M11 Mutant Myotubes (*n* = 57)	M11+mM06 Hybrid Myotubes (*n* = 34)	M06 Control Myotubes (*n* = 41)
MFI %	28.6 ± 12.5	29.3 ± 11.6	35.4 ± 12.1	41.4 ± 9.7	36.6 ± 14.4
Nr of nuclei/myotube	5.8 ± 2.8	8.0 ± 4.2 *	8.8 ± 6.1	12.4 ± 8.8 *	8.8 ± 6.2
Sarcoplasm volume/myotube	(2.53 ± 1.10) × 10^4^	(4.5 ± 2.3) × 10^4^ *	(4.2 ± 2.5) × 10^4^	(4.0 ± 2.3) × 10^4^	(5.2 ± 4.5) × 10^4^
Sarcoplasm volume/myotube nucleus	(4.8 ± 0.2) × 10^3^	(5.9 ± 2.0) × 10^3^ *	(5.8 ± 3.8) × 10^3^	(3.9 ± 1.9) × 10^3^ *	(5.6 ± 2.3) × 10^3^

All values are mean ± SD; * significantly changed in hybrid compared to non-hybrid myotubes based on *t*-test, *p* < 0.05; Volumes are µm^3^.

**Table 2:** Comparison of mitochondrial parameters in non-hybrid myotubes.

	M02 Mutant Myotubes	M11 Mutant Myotubes	M06 Control Myotubes	ANOVA *p*-Value
Mitochondrial volume per sarcoplasm volume	0.27 ± 0.05	0.27 ± 0.04	0.28 ± 0.03	0.37
Mitochondrial membrane potential (A.U./µm^3^)	2.0 ± 0.7 × 10^4^	2.0 ± 0.5 × 10^4^	2.2 ± 0.5 × 10^4^	0.36
Mitochondrial object number per 1000 µm^3^ of sarcoplasm	3.2 ± 1.1	3.0 ± 0.9	3.1 ± 0.8	0.36

All values are mean ± SD.

**Table 3:** Analysis of mitochondrial parameters in hybrid myotubes compared to mutant myotubes.

	M02 Mutant Myotubes	M02+mM06 Hybrid Myotubes	FC	M11 Mutant Myotubes	M11+mM06 Hybrid Myotubes	FC
Mito volume/myotube	6.88 ± 3.20 × 10^3^	11.62 ± 6.38 × 10^3^	1.70 *	11.61 ± 7.31 × 10^3^	11.13 ± 6.75 × 10^3^	0.95
Mito volume/sarcoplasm volume	0.28 ± 0.05	0.26 ± 0.03	0.92	0.27 ± 0.04	0.28 ± 0.03	1.02
Mito volume/nucleus	1.28 ± 0.43 × 10^3^	1.52 ± 0.47 × 10^3^	1.15 *	1.53 ± 0.98 × 10^3^	1.06 ± 0.47 × 10^3^	0.70 *
Mito intens/myotube	5.38 × 10^8^ ± 2.72 × 10^8^	8.45 × 10^8^ ± 4.39 × 10^8^	1.57 *	8.70 × 10^8^ ± 5.98 × 10^8^	8.05 × 10^8^ ± 4.47 × 10^8^	0.93
Mito intens/sarcoplasm volume	2.02 ± 0.72 × 10^4^	1.93 ± 0.44 × 10^4^	0.95	2.02 ± 0.5 × 10^4^	2.10 ± 0.51 × 10^4^	1.03
Mito intens/nucleus	9.8 ± 0.36 × 10^7^	1.11 ± 0.37 × 10^8^	1.13	1.11 ± 7.27 × 10^8^	8.01 ± 4.15 × 10^7^	0.72 *
Mito objects/myotube	80.76 ± 32.91	124.55 ± 59.09	1.55 *	116.93 ± 63.78	126.35 ± 71.40	1.07
Mito objects/sarcoplasm volume	0.33 ± 0.11 × 10^−2^	0.30 ± 0.07 × 10^−2^	0.90	0.30 ± 0.09 × 10^−2^	0.32 ± 0.07 × 10^−2^	1.09
Mito objects/nucleus	16 ± 7	17 ± 6	1.10	16 ± 9	12 ± 6	0.76 *

* significantly changed, *t*-test *p* < 0.05; FC = fold change hybrid vs. non-hybrid myotubes; Mito = mitochondrial; Mito. Intens = Mitochondrial intensity based on TMRM signal intensity (A.U.); All volumes shown in table are µm^3^.

## Data Availability

The original contributions presented in this study are included in the article/Supplementary Material. Further inquiries can be directed to the corresponding author.

## Supplementary Materials

The following supporting information can be downloaded at https://www.mdpi.com/article/10.3390/ijms27031357/s1.

## References

[1] Wai T., Ao A., Zhang X., Cyr D., Dufort D., Shoubridge E.A. (2010). The role of mitochondrial DNA copy number in mammalian fertility. Biol. Reprod..

[2] Trenell M.I., Sue C.M., Kemp G.J., Sachinwalla T., Thompson C.H. (2006). Aerobic exercise and muscle metabolism in patients with mitochondrial myopathy. Muscle Nerve.

[3] Jeppesen T.D., Schwartz M., Frederiksen A.L., Wibrand F., Olsen D.B., Vissing J. (2006). Muscle phenotype and mutation load in 51 persons with the 3243A>G mitochondrial DNA mutation. Arch. Neurol..

[4] Ahmed S.T., Craven L., Russell O.M., Turnbull D.M., Vincent A.E. (2018). Diagnosis and Treatment of Mitochondrial Myopathies. Neurotherapeutics.

[5] van Tienen F., Zelissen R., Timmer E., van Gisbergen M., Lindsey P., Quattrocelli M., Sampaolesi M., Mulder-den Hartog E., de Coo I., Smeets H. (2019). Healthy, mtDNA-mutation free mesoangioblasts from mtDNA patients qualify for autologous therapy. Stem Cell Res. Ther..

[6] van Tienen F.H.J., Hoeijmakers J.G.J., van der Leij C., Timmer E., Wanders N., Lindsey P.J., Yi F., Lin F., Kortekaas S.P.M., Roelofs H. (2025). Intra-arterial transplantation of autologous mesoangioblasts in m.3243A>G mutation carriers is safe—First phase I/II human clinical study. Mol. Ther..

[7] Zelissen R., Ahmadian S., Montilla-Rojo J., Timmer E., Ummelen M., Hopman A., Smeets H., van Tienen F. (2023). Fusion of Wild-Type Mesoangioblasts with Myotubes of mtDNA Mutation Carriers Leads to a Proportional Reduction in mtDNA Mutation Load. Int. J. Mol. Sci..

[8] Ahmadian S., Lindsey P.J., Smeets H.J.M., van Tienen F.H.J., van Zandvoort M. (2024). Spinning Disk Confocal Microscopy for Optimized and Quantified Live Imaging of 3D Mitochondrial Network. Int. J. Mol. Sci..

[9] Hayashi J., Ohta S., Takai D., Miyabayashi S., Sakuta R., Goto Y., Nonaka I. (1993). Accumulation of mtDNA with a mutation at position 3271 in tRNA(Leu)(UUR) gene introduced from a MELAS patient to HeLa cells lacking mtDNA results in progressive inhibition of mitochondrial respiratory function. Biochem. Biophys. Res. Commun..

[10] Uziel G., Carrara F., Granata T., Lamantea E., Mora M., Zeviani M. (2000). Neuromuscular syndrome associated with the 3291T→C mutation of mitochondrial DNA: A second case. Neuromuscul. Disord..

[11] Stephan T., Roesch A., Riedel D., Jakobs S. (2019). Live-cell STED nanoscopy of mitochondrial cristae. Sci. Rep..

[12] Ren W., Ge X., Li M., Sun J., Li S., Gao S., Shan C., Gao B., Xi P. (2024). Visualization of cristae and mtDNA interactions via STED nanoscopy using a low saturation power probe. Light Sci. Appl..

[13] Chan D.C. (2006). Mitochondria: Dynamic organelles in disease, aging, and development. Cell.

[14] Chal J., Pourquié O. (2017). Making muscle: Skeletal myogenesis in vivo and in vitro. Development.

[15] Zhang C., Huang V.H., Simon M., Sharma L.K., Fan W., Haas R., Wallace D.C., Bai Y., Huang T. (2012). Heteroplasmic mutations of the mitochondrial genome cause paradoxical effects on mitochondrial functions. FASEB J..

[16] Hertig D., Felser A., Diserens G., Kurth S., Vermathen P., Nuoffer J.M. (2019). Selective galactose culture condition reveals distinct metabolic signatures in pyruvate dehydrogenase and complex I deficient human skin fibroblasts. Metabolomics.

[17] Haun C.T., Vann C.G., Roberts B.M., Vigotsky A.D., Schoenfeld B.J., Roberts M.D. (2019). A Critical Evaluation of the Biological Construct Skeletal Muscle Hypertrophy: Size Matters but So Does the Measurement. Front. Physiol..

[18] Khodabukus A. (2021). Tissue-Engineered Skeletal Muscle Models to Study Muscle Function, Plasticity, and Disease. Front. Physiol..

[19] Jayaprakash A.D., Benson E.K., Gone S., Liang R., Shim J., Lambertini L., Toloue M.M., Wigler M., Aaronson S.A., Sachidanandam R. (2015). Stable heteroplasmy at the single-cell level is facilitated by intercellular exchange of mtDNA. Nucleic Acids Res..

[20] Legros F., Lombes A., Frachon P., Rojo M. (2002). Mitochondrial fusion in human cells is efficient, requires the inner membrane potential, and is mediated by mitofusins. Mol. Biol. Cell.

[21] Schouten H.C., Hopman A.H., Haesevoets A.M., Arends J.W. (1995). Large-cell anaplastic non-Hodgkin’s lymphoma originating in donor cells after allogenic bone marrow transplantation. Br. J. Haematol..

[22] Iannetti E.F., Smeitink J.A., Beyrath J., Willems P.H., Koopman W.J. (2016). Multiplexed high-content analysis of mitochondrial morphofunction using live-cell microscopy. Nat. Protoc..

