# Supplementary material for: Fusion Between Control Mesoangioblasts and mtDNA-Mutant Myotubes Preserves Myotube Morphology and Mitochondrial Network Organization
Source: Int J Mol Sci. 2026 Jan 29;27(3):1357. doi: 10.3390/ijms27031357 (PMC12897884; doi:10.3390/ijms27031357)
Supplement: Supplementary file 1 [file ijms-27-01357-s001.zip › ijms-4082099-supplementary.pdf]

Supplementary table S1. Descriptive data of the parameters for non-hybrid myotubes.

**Parameter**	**M02 (n=25)**	**M11 (n=57)**	**M06 (n=41)**
Sum Mitochondrial Volume (μm^3) (min-max)	4.0-19.9 x10^3	3.8-36.0 x10^3	3.8-26.8 x10^3
Sum Mitochondrial Volume (μm^3) (Mean ± SD)	6.9 ± 3.2 x10^3	11.6 ± 7.3 x10^3	11.6 ± 5.6 x10^3
Sum Mean Intensity (A.U.)(min-max)	9.5-58.2 x10^3	12.4-111.4 x10^3	9.1-120.3 x10^3
Sum Mean Intensity (A.U.)(Mean ± SD)	26.5 ± 10.6 x10^3	41.0 ± 20.5 x10^3	41.0 ± 21.4 x10^3
Sum Intensity (A.U.) (min-max)	2.1×10^8-1.5×10^9	1.8×10^8-0.9×10^9	2.8×10^8-2.0×10^9
Sum Intensity (A.U.) (Mean ± SD)	5.4×10^8±2.7×10^8	8.7×10^8±6.0×10^8	8.7×10^8 ± 4.3×10^8
Unique Mitochondria Count (min-max)	27-171	30-346	37-335
Unique Mitochondria Count (Mean ± SD)	80.8 ± 32.9	116.9 ± 63.8	123.3 ± 61.8
Cell Volume (μm^3) (min-max)	0.1 -0.7 x10^5	0.2-1.3 x10^5	0.1-1.2 x10^5
Cell Volume (μm^3) (Mean ± SD)	2.8 ± 1.1 x10^4	4.6 ± 2.6 x10^4	4.5 ± 2.2 x10^4
Nucleus Number (min-max)	3-15	3-32	3-36
Nucleus Number (Mean ± SD)	5.8 ± 2.8	8.8 ± 6.1	8.6 ± 6.3

Supplementary Table S2. Descriptive data of the parameters of hybrid myotubes.

**Parameter**	**M02+mM06 (n=38)**	**M11+mM06 (n=34)**
Sum Mitochondrial Volume (μm^3) (min-max)	3.9-24.8x10^3	4.8-36.8 x10^3
Sum Mitochondrial Volume (μm^3) (Mean ± SD)	11.6 ± 6.4 x10^3	11.1 ± 6.8 x10^3
Sum Mean Intensity (A.U.) (min-max)	18.3-85.1 x10^3	13.6-109.8 x10^3
Sum Mean Intensity (A.U.) (Mean ± SD)	42.4 ± 19.4 x10^3	41.8 ± 20.2 x10^3
Sum Intensity (A.U.) (min-max)	2.6×10^8-1.7×10^9	3.0×10^8-2.0×10^9
Sum Intensity (A.U.) (Mean ± SD)	$8.4\times10^8 \pm 4.4\times10^8$	$8.1\times10^8 \pm 4.5\times10^8$
Unique Mitochondria Count (min-max)	49-303	41-395
Unique Mitochondria Count (Mean ± SD)	124.6 ± 59.1	126.4 ± 71.4
Cell Volume (µm3) (min-max)	0.2-1.1 x10^5	0.2-1.5x10^5
Cell Volume (A.U.) (Mean ± SD)	4.8 ± 2.5 x10^4	4.6 ± 2.6 x10^4
Nucleus Number (min-max)	3-18	4-39
Nucleus Number (Mean ± SD)	8.0 ± 4.2	12.4 ± 8.7
Y Chromosome (min-max)	1-10	1-12
Y Chromosome (Mean ± SD)	2.7 ± 2.0	3.0 ± 2.5

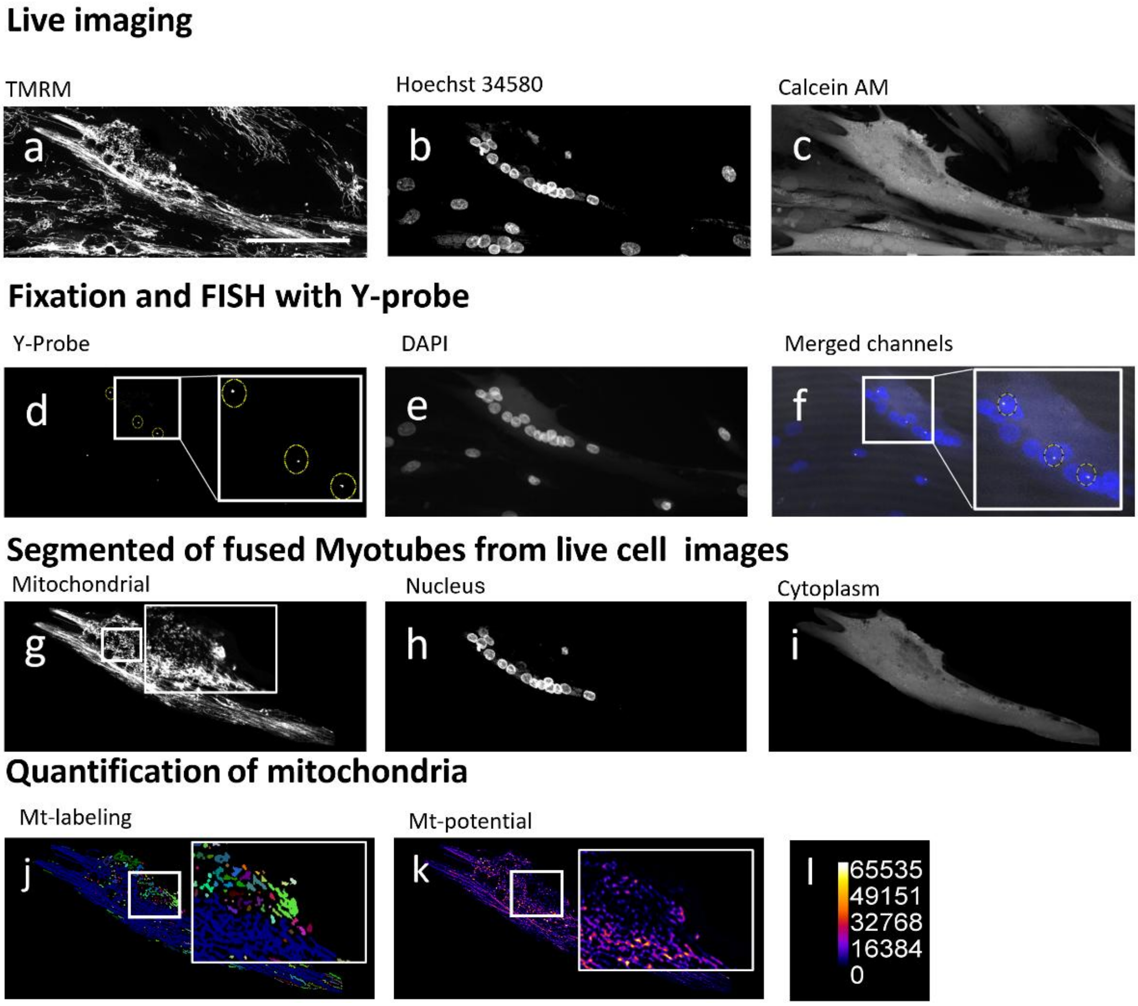

Supplementary figure S1. Detection of fused control mesoangioblasts with mutated myotubes. a-c) Live cell imaging using SDCM of mutated myotubes fused with control mesoangioblasts **a)** mitochondrial network stained with TMRM probe, **b)** nuclei stained with Hoechst 34580, **c)** cell stained with Calcein AM; **d-f)** FISH imaging of Y-chromosome **d)** Y chromosome probe (DYZ3) conjugated to Y-ATTO 550 was visualized as white dots, highlighted by circle **e)** nuclei stained with DAPI and same field of view captured as with live cell imaging, **f)** merged image of Y-chromosome probe and nucleus channel, arrows shows the number of nucleus containing Y-chromosome; **g-i**) segmentation of fused myotube of interest from live cell imaging, **g)** segmented mitochondrial network stained with TMRM probe, **h)** segmented nucleus stained with Hoechst 34580, **i)** segmented cell stained with Calcein AM, **j**) mitochondrial classification, each color representing a separate mitochondrial object that is not connected to the rest of the mitochondrial network, and **k**) qualitative image of intensity of TMRM probe representative of mitochondrial, membrane potential, the brighter area having higher membrane potential , **l)** calibration bar for k. Scale bar is 100μm.

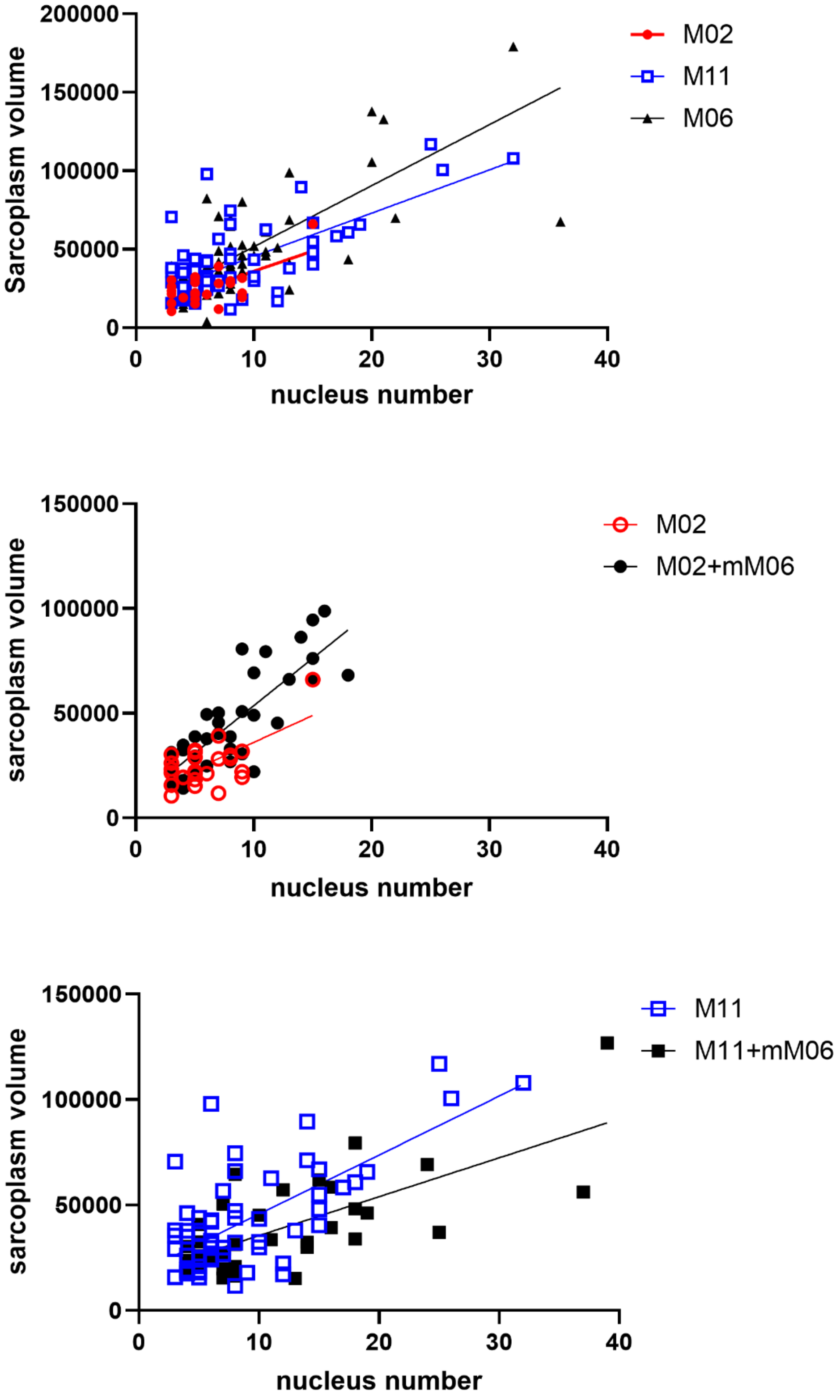

Supplemental figure S2. Relationship between sarcoplasm volume and number of nuclei per myotube.
